# Nature of selection varies on different domains of IFI16-like PYHIN genes in ruminants

**DOI:** 10.1186/s12862-018-1334-7

**Published:** 2019-01-17

**Authors:** Sushil Kumar, Jatinder Singh Chera, Ashutosh Vats, Sachinandan De

**Affiliations:** Animal Genomics Laboratory, Animal Biotechnology Centre, National Dairy Research Institute, Karnal, Haryana 132001 India

**Keywords:** PAMP, DAMP, PRR, IFI16, IFI16-like, PYHIN, PYD, HIN, Inter-domain, Selection

## Abstract

**Background:**

ALRs (AIM2-like Receptors) are germline encoded PRRs that belong to *PYHIN* gene family of cytokines, which are having signature N-terminal PYD (Pyrin, PAAD or DAPIN) domain and C-terminal HIN-200 (hematopoietic, interferon-inducible nuclear protein with 200 amino acid repeat) domain joined by a linker region. The positively charged HIN-200 domain senses and binds with negatively charged phosphate groups of single-stranded DNA (ssDNA) and double-stranded DNA (dsDNA) purely through electrostatic attractions. On the other hand, PYD domain interacts homotypically with a PYD domain of other mediators to pass the signals to effector molecules downwards the pathways for inflammatory responses. There is remarkable inter-specific diversity in the numbers of functional *PYHIN* genes e.g. one in cow, five in human, thirteen in mice etc., while there is a unique loss of *PYHIN* genes in the bat genomes which was revealed by Ahn et al. (2016) by studying genomes of ten different bat species belonging to sub-orders *yinpterochiroptera* and *yangochiroptera*. The conflicts between host and pathogen interfaces are compared with “Red queen’s arms race” which is also described as binding seeking dynamics and binding avoidance dynamics. As a result of this never-ending rivalry, eukaryotes developed PRRs as antiviral mechanism while viruses developed counter mechanisms to evade host immune defense. The *PYHIN* receptors are directly engaged with pathogenic molecules, so these should have evolved under the influence of selection pressures. In the current study, we investigated the nature of selection pressure on different domain types of *IFI16-like* (*IFI16-L*) *PYHIN* genes in ruminants.

**Results:**

Three transcript variants of the *IFI16-like* gene were found in PBMCs of ruminant animals-water buffalo, zebu cattle, goat, and sheep. The *IFI16-like* gene has one N-terminal PYD domain and one C-terminal HIN-200 domain, separated by an inter-domain linker region. HIN domain and inter-domain region are positively selected while the PYD domain is under the influence of purifying selection.

**Conclusion:**

Herein, we conclude that the nature of selection pressure varies on different parts (PYD domain, HIN domain, and inter-domain linker region) of *IFI16-like PYHIN* genes in the ruminants. This data can be useful to predict the molecular determinants of pathogen interactions.

**Electronic supplementary material:**

The online version of this article (10.1186/s12862-018-1334-7) contains supplementary material, which is available to authorized users.

## Background

Immune defense mechanism in mammalian physiology is a highly coordinated process, which activates various mechanisms for local and systemic immunity. The first line of defense, the innate immune system, relies on Pattern Recognition Receptors (PRRs) to identify Pathogen-Associated Molecular Patterns (PAMPs) and Damage-Associated Molecular Patterns (DAMPs) for immune response generation [1–4]. PAMPs and DAMPs are the signature molecules of pathogens and damaged host cells respectively. Five categories of PRRs have been characterized in the mammalian cells which can be divided into two groups based on whether they are membrane-bound or free. Membrane-bound PRRs are *Toll-like Receptors (TLRs)* and *C-type Lectin Receptors (CLRs)*. PRRs which reside and function inside the cellular compartments include *NOD-like Receptors (NLRs)*, *RIG1-like receptors (RLRs)* and *AIM2-like receptors (ALRs)* [5–7]. These PRRs work in harmony to identify various forms of PAMPs and DAMPs (nucleic acids, proteins, lipids, and carbohydrates) inside the cell and initiate signaling cascade which leads to the expression of interferons and proinflammatory cytokines [8]. Any impairment of this molecular harmony can disturb physiological homeostasis and lead to dire consequences in the host.

A DNA molecule can be categorized in either DAMPs (if it comes from the damaged host cell or mitochondria) or PAMPs (if it originates from intracellular or extracellular pathogens). A number of proteins have been proposed as putative PRRs for DNA. These PRRs include- *Absent in Melanoma 2* (*AIM2*), *Interferon-γ inducible protein 16* (*IFI16*), cyclic *GMP- AMP synthase* (*cGAS*), *Z-DNA binding protein1* (*DAI*), *Toll-like Receptor 9* (*TLR9*) and *RNA pol III*. The presence of so many DNA sensors is still an unraveled mystery [9]. Among all these, *IFI16* and *AIM2*, which come under the category of *ALRs*, function in the recognition of viral DNA inside the cellular compartments [5, 10, 11]. *ALRs* are germline encoded PRRs that belong to the *PYHIN* gene family of cytokines, which have signature N-terminal PYD (Pyrin, PAAD or DAPIN) domain and a C-terminal HIN-200 (hematopoietic, interferon-inducible nuclear protein with 200 amino acid repeat) domain joined by a linker region [12, 13]. The positively charged HIN-200 domain senses and binds with the negatively charged phosphate groups of single-stranded DNA (ssDNA) and double-stranded DNA (dsDNA) purely through electrostatic attractions. On the other hand, the PYD domain interacts homotypically with PYD domains of other mediators to pass the signals to the effector molecules downstream of the pathways for inflammatory responses [14]. The role of inter-domain linker region is proposed in the alignment of PYD and HIN-200 domains for properly achieving their respective 3D structures and functions.

The syntenic *PYHIN* gene cluster region among mammalian species is variable in its size, number and gene arrangements. In addition, it also possesses signature border markers of *cell adhesion molecule 3 (CADM3)* genes on one end, and *olfactory receptors* and *spectrin alpha chain (SPTA1)* genes on the other end in all mammals [15]. This genomic region is believed to have evolved through multiple events of gene duplications, recombination, and species-specific family expansions/losses. There is also a remarkable inter-specific diversity in the numbers of functional *PYHIN* genes which varies with one in *cow*, five in *human*, thirteen in *mouse* (Additional file 1), and a unique loss of *PYHIN* genes in the genomes of ten different *bat* species belonging to sub-orders yinpterochiroptera and yangochiroptera [15–17].

The *PYHIN* receptors directly engage with pathogenic molecules, they may have evolved under selective pressure from the pathogens [18, 19]. The conflicts between host and pathogen interfaces are compared with “Red queen’s arms race” which is also described as binding seeking dynamics and binding avoidance dynamics [20]. According to this hypothesis, host restriction factors adapt positively to binding to viral factors and on the other hand, due to the course of evolution, viruses also develop mechanisms to avoid host immune responses. As a result of this never-ending rivalry, eukaryotes developed PRRs as antiviral mechanism while viruses developed counter mechanisms to evade host immune defense. For example, *HSV-1* encoded immediate-early protein *ICP0*, an *E3 ubiquitin ligase*, binds and directs the proteasomal degradation of *IFI16*. Another protein *pUL83* encoded by *HCMV* binds with PYD domain of *IFI16* and blocks its multimerization.

In the current study, we investigated the nature of selection pressure on different domain types of *IFI16 like (IFI16-L) PYHIN* genes in ruminants of the order Artiodactyla; all members of which are predicted to possess single functional *PYHIN* gene in their genomes. The inflammasome forming *AIM2* is present as the pseudogene in the genomes of the ruminant animals. This gene became inactive due to accumulations of deletion, frame-shift and stop codons [16]. The *ALR* family member *IFI16* is thought to act as an important innate immune PRR for viral DNA inside cytoplasmic and nuclear compartments of the cell. It detects and binds with viral dsDNA as well as viral ssDNA stem-loop structures [21]. Various studies have shown direct engagements of *IFI16* with *herpesviruses* in cellular compartments of a variety of human cell types [22]. *Bovine herpesviruses* (DNA viruses) are responsible for a variety of disease conditions in both domestic and wild ruminants and cause a severe economic loss in the form of abortion, reduced production and impaired work abilities [23, 24]. *IFI16* induces inflammasome and *interferon-β* through *PYCARD* and *STING* mediated pathways respectively [4, 25–27]. It is also reported to acts as viral restriction factor through epigenetic modifications [28–30]. *Herpesviruses* cause both lytic infections and latent infections. *IFI16* has a critical role in herpesviral latency, which makes it a potential therapeutic target against herpesviral infections [31]. The characterization and evolutionary analysis of *IFI16-L* gene in ruminant animals will help to identify residues which directly engage with viruses and will give insights into molecular markers of disease resistant/susceptibility against DNA viruses including *herpesviruses* [32]. This will further be helpful in designing of innate immunologicals and vaccine strategies [33].

Herein, we conclude that the nature of selection pressure varies on different parts (PYD domain, HIN domain, and the inter-domain linker region) of *IFI16-like PYHIN* genes in the ruminants.

## Results

### *IFI16-like PYHIN* genes have multiple alternatively spliced transcript variants

**Fig. 1:**
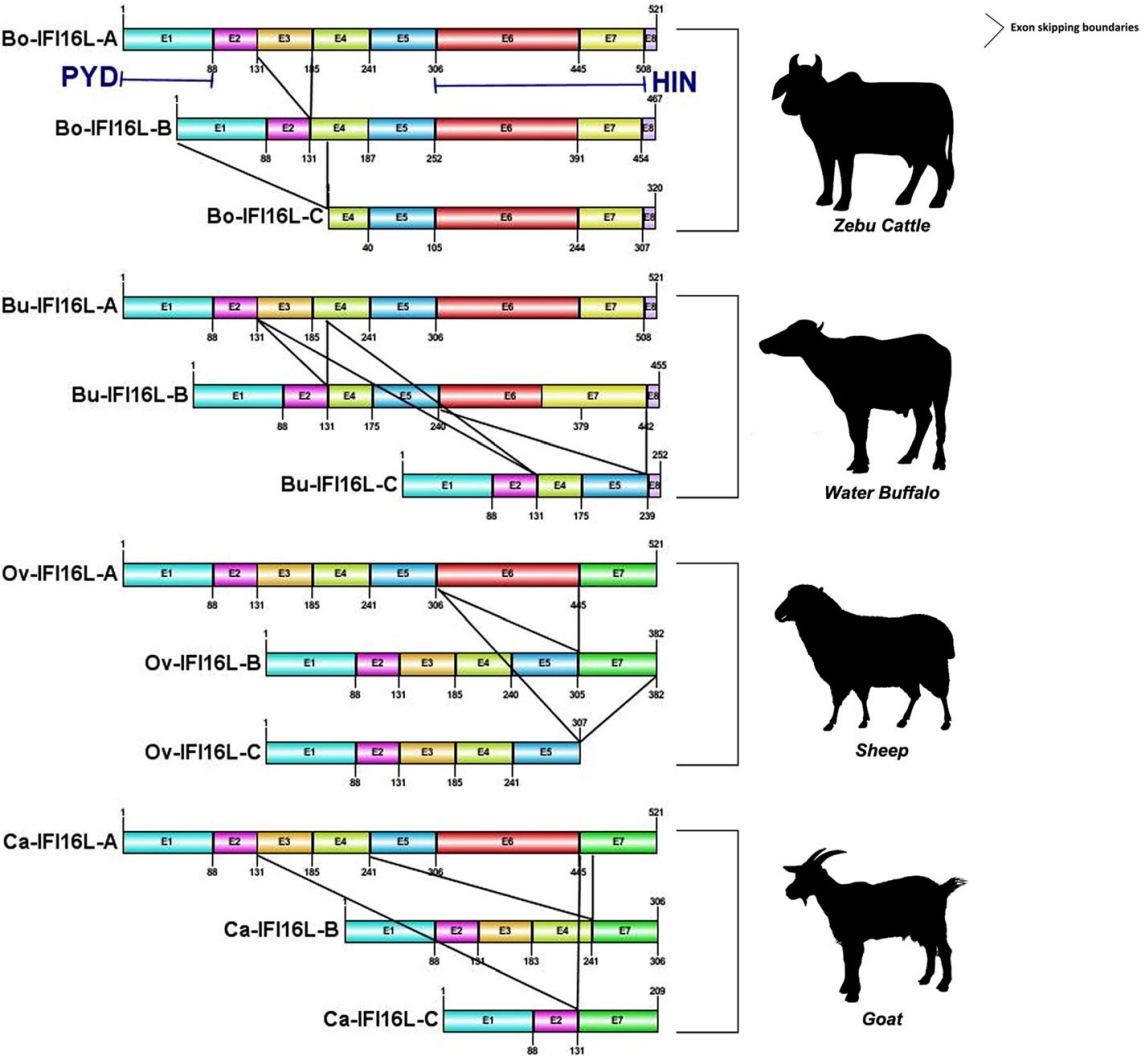
Exon skipping of different transcript variants of *IFI16-like PYHIN* genes. The three transcript variants of different lengths of *IFI16-like* genes in *buffalo*, *zebu cattle*, *sheep* and *goat* were studied. The illustration depicts exon skipping among transcript variants of *IFI16-like* genes. In case of *buffalo* and *cow*, there are eight exons while in the case of sheep and goat, 8th exon is fused with 7th exon

In the present study, the *IFI16-like ALR* gene sequences were selectively amplified, cloned and sequenced from *Indian cattle* (*Bos indicus), water buffalo (Bubalus bubalis), goat (Capra hircus)* and *sheep (Ovis aries*). The transcript variant ‘C’ in *buffalo* and *sheep*; and ‘B’ transcript of *goat* lacks HIN domain while the cattle transcript variant ‘C’ lacks PYD domain and a putative nuclear localization signal (Fig. 1).

### *IFI16-like ALR* is a shuttle protein like human ALR IFI16

**Fig. 2:**
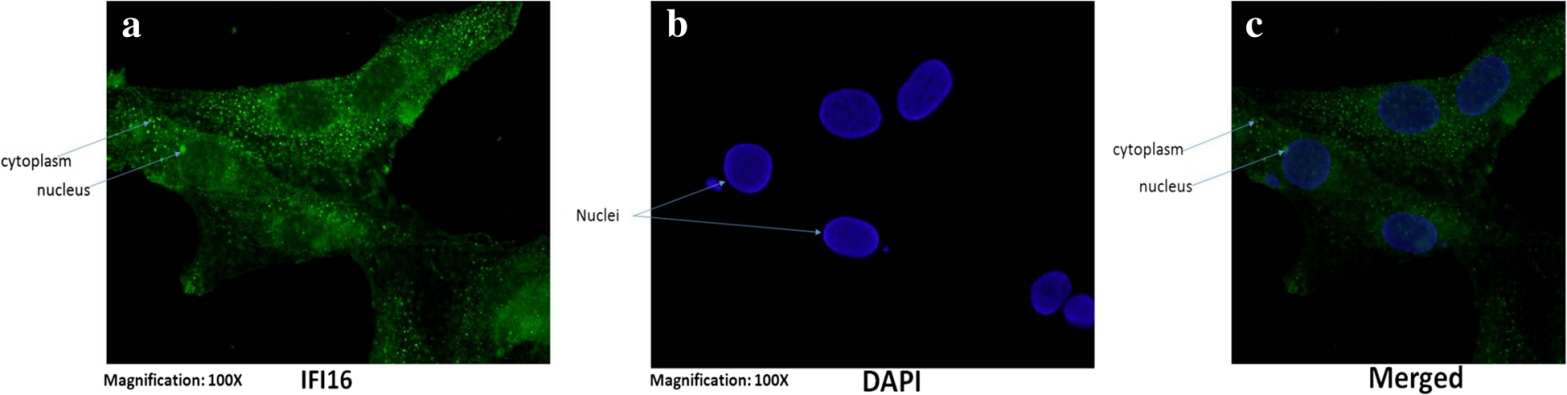
Intra-cellular localization of ruminant *IFI16-L* proteins. Immuno-cytochemistry was carried out for *IFI16-L* proteins in the cultured buffalo fetal fibroblast cells (**a**) Green fluorescence showing the localization of *IFI16-L* protein; (**b**) Fluorescence showing nuclei of the fetal fibroblast cells stained with DAPI; (**c**) Image formed by merging A and B. The *IFI16-L* protein was found to co-localize in both, the nucleus as well as the cytoplasm

Through Immuno-cytochemistry in buffalo fibroblasts, we found that *IFI16-like PYHIN* protein was localized in both nuclear and cytoplasmic compartments (Fig. 2). For shuttling between nucleus and cytoplasm, it contains a nuclear localization signal (NLS) at its N-terminal which is partially fused with PYD domain (Fig. 1). Single putative NLS motif found in Buffalo (82 - LRKEKLKVLKKSKAK – 96), sheep (83 - RKEKLKAIRRKKVK – 96) and goat (83 - RKEKLKAIKKNKAK – 96) while two putative NLS motif sequences (82 - LRKEKLKVIKKNKAK – 96, 131 - KKKRTTKTNESKRR – 144) were identified in the *IFI16-L* gene of zebu cattle.

### Phylogenetic analysis of *IFI16-L* gene domains reveals their evolutionary relatedness to *human IFI16* domains

**Fig. 3:**
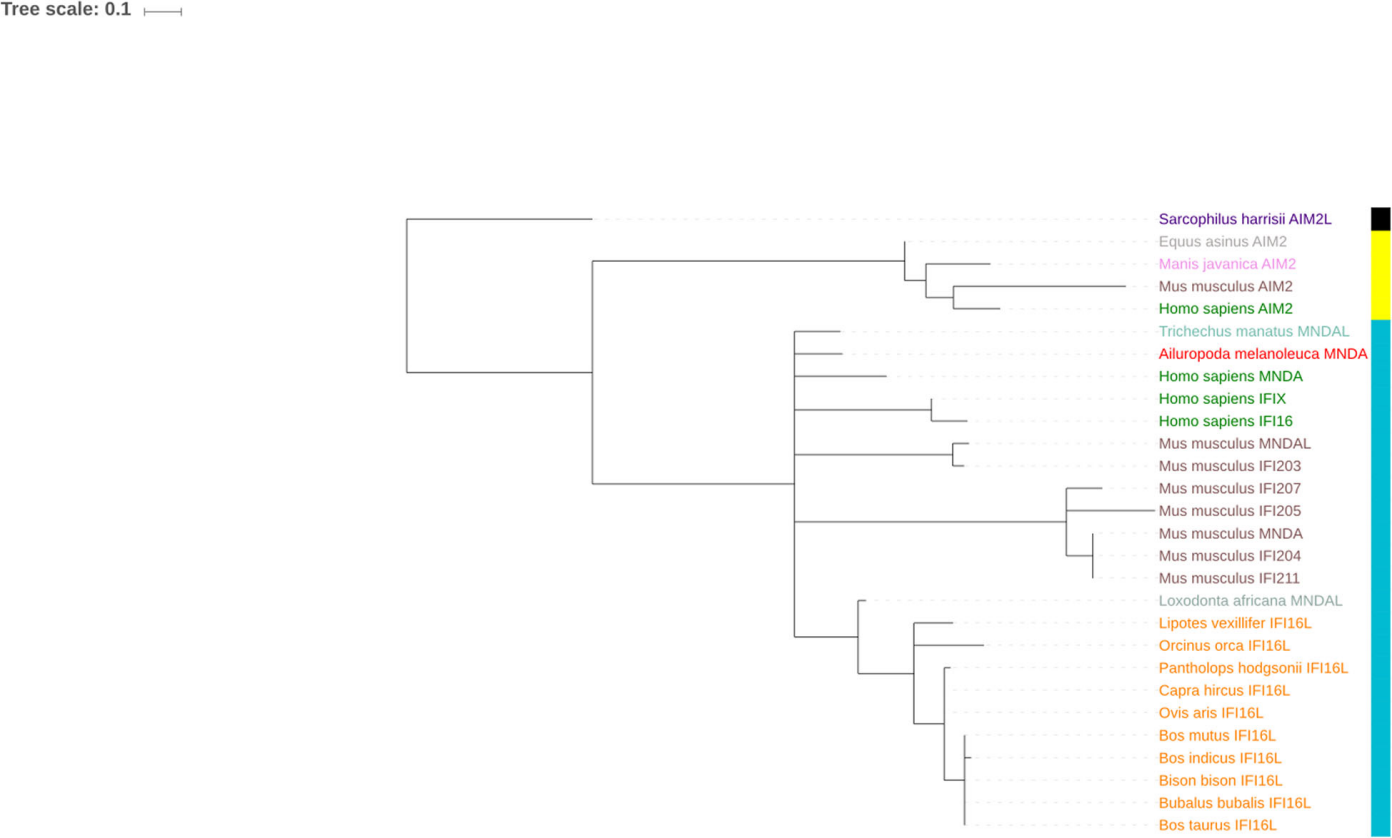
ML phylogenetic tree of HIN domains from different mammalian orders rooted with marsupial sequence. ML phylogenetic tree is rooted on marsupial *Sarcophilus harrisii*, which is taken as the outgroup. All nodes having bootstrap values of < 50% were collapsed. Tree was visualized using iTOL. Names of the animals belonging to the same mammalian order have same color. The color-strip represent the type of HIN domain of corresponding PYHIN gene- HINa (turquoise) HINb (yellow), HINc (violet) and HINd (black)

**Fig. 4:**
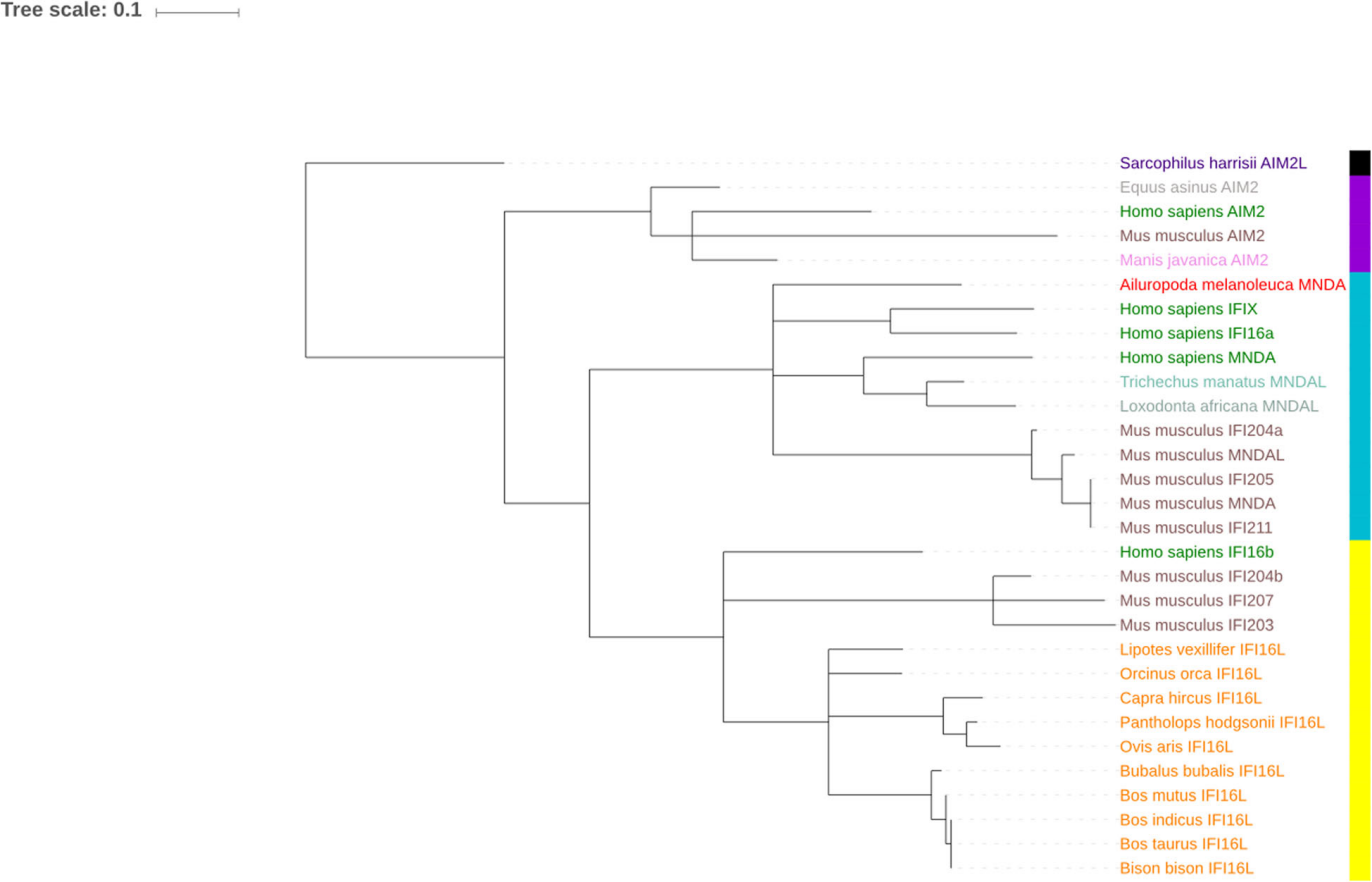
ML phylogenetic tree of PYD domains from different mammalian orders rooted with marsupial sequence. ML phylogenetic tree is rooted on marsupial *Sarcophilus harrisii*, which is taken as the outgroup. All nodes having bootstrap values of < 50% were collapsed. Tree was visualized using iTOL. Names of the animals belonging to the same mammalian order have same color. The color-strip represent the type of PYD domain of corresponding *PYHIN* gene- IFI (non-AIM2) PYD domain (turquoise), AIM2 PYD (Yellow) and marsupial PYD (black)

**Fig. 5:**
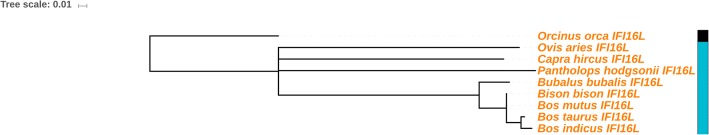
ML phylogenetic tree of inter-domains (ID) from ruminants *IFI16-L* gene, rooted with whale sequence. ML phylogenetic tree is rooted on cetacean *Orcinus orca* (whale), which is taken as the outgroup. All nodes having bootstrap values of < 50% were collapsed. Tree was visualized using iTOL. Names of the animals belonging to the same mammalian order (Artiodactyla) have same color. The color-strip represent the type of inter-domain of corresponding ruminant ID (turquoise), and whale ID (black)

The sequences of ALRs from different species which represented different mammalian orders were collected from NCBI for phylogenetic analysis. The inter-domain linker region between PYD and HIN domains is highly variable, therefore, phylogenetic analysis of whole genes and of the inter-domain (ID) linker region alone in unrelated species might produce misleading results [15, 16]. So, all the domain sequences and inter-domain sequences were used separately for the alignment and analysis. Three separate phylograms were generated based on their amino acid alignments by maximum likelihood (ML) approaches (Figs. 3, 4 and 5). Phylogenetic analyses were conducted by “one click” mode at *Phylogeny.fr* suite of programs [34, 35] using PhyML 3.0 with 100 bootstrap replicates and the trees were drawn using TreeDyn 198.3 software. Trees of domains i.e. PYD and HIN were rooted with the marsupial Tasmanian devil (*Sarcophilus harrisii*) and their topologies were found to be congruent with the previously reported phylograms of HIN and PYD domains. The phylogenetic tree of inter-domain linker region was rooted on the whale *Orcinus orca*.

The HIN domain ML phylogram produced three clades of placental mammal sequences based on their HIN domain types i.e. HINa, HINb and HINc while the fourth clade of the marsupial sequence has HINd domain which aligns separately. The HIN domains of *IFI16-L ALRs* among species members of the order Artiodactyla (which also include ruminants), align in the clade of HINb domain along with the HINb domain of human *IFI16*.

On the other hand, the PYD domain phylogram shows different tree topology compared to the HIN domain tree topology and it produced two separate clades among placental mammals while the third clade is made of the marsupial sequence. One clade of placental mammals formed by *AIM2-like PYHIN* genes while another clade is formed by the rest of the interferon-inducible *PYHIN* genes including ruminant *IFI16-L*. The phylogram of inter-domain sequences formed a single clade of ruminant animals.

### Nature of selection varies along the different domain regions of ruminant *IFI16-like PYHIN* gene

To explore variations in selective pressures among the PYD domain, HIN domain, and inter-domain, various statistical models as implemented in Datamonkey web server, were employed. The full length nucleic acid ORF sequences of selected ruminants were divided into three parts, PYD domain, HIN domain and the inter-domain. Since recombining sequences can give misleading results in phylogenetic analysis [15, 16], the DSS method [36] was used as implemented in the TOPALi program [37]. No recombination breakpoints were found using this method. Recombination breakpoints were further checked in multiple sequence alignments by GARD (Genetic Analysis for Recombination Detection) algorithm [38]. The GARD analyses revealed the presence of one putative recombination breakpoint in the HIN domain alignment, which was found non-significant to produce a topological incongruence by the KH (Kishino–Hasegawa test, at *P* = 0.1) test.

The HyPhy software package at datamonkey web server [39] was subsequently used to analyze the sequences. With the user-friendly graphical interfaces, new fast methods and multi-tasking abilities, HyPhy provides better analysis power to users in comparison of PAML software package [40–46]. The web-server version of HyPhy i.e. Datamonkey provides additional advantages. With the use of the codon model selection (CMS) module, HKY85 was found as the best model for the statistical analysis of all the aligned sequences. PARRIS (a PARtitioning approach for Robust Inference of Selection) test [47] from HyPhy package (implemented in Datamonkey web server) was used to find the positive selection pressure. This test is similar to the M2a versus M1a test (PAML) but it (PARRIS) also considers synonymous substitution rate variations (dS). We found the evidences of positive selection (at *p* < 0.1threshold) in HIN domain (*p*-value = 0.047) and the inter-domain linker region (p-value = 0.080) while PARRIS test inferred no evidence of positive selection in the PYD domain (*p*-value = 0.999) at *p* < 0.1 threshold (see Additional file 2). A second-round analysis was performed which was aimed at the detection of selection at individual codons. The nature of selection at individual codon can highlight the residues interacting with pathogenic molecules. As the aim was to find out the individual sites under selections, so models like aBSREL, which provides information about lineage specific selection were not taken into considerations. The fixed effect likelihood (FEL) method and fast unconstrained Bayesian approximation method (FUBAR) [48] were applied to all the alignments. FUBAR use a Bayesian approach and produces similar information to FEL, but it is more powerful and fast in comparison to FEL [48–50]. Both FEL and FUBAR analysis (*p*-value < 0.1 and posterior probability > 0.9 respectively) identified codon sites that evolved under the influence of pervasive diversifying selection and pervasive purifying selection. A total of ten codons in the inter-domain region and five codons in the HIN domain were identified to be evolved under pervasive diversifying selection (Table 1) but no codon was found to be positively selected in the PYD domain. Codons evolved under pervasive purifying selection were six in PYD domain, seven in HIN domain and three in inter-domain linker region (Table 1). Mixed Effect Likelihood Model (MEME) [40] was also applied to all the aligned sequences in order to explore the episodic diversifying selection on the individual codons. Three codons in the HIN domain and only one codon in the inter-domain region were observed through this analysis. On the contrary, no episodic diversifying selection was observed in the PYD domain (at the *p* < 0.05 level). These results make clear demarcation about the type of selection pressure on different parts of *IFI16-L* sequences.

**Table 1:** Positively and negatively selected codon sites in different domains of *IFI16-like PYHIN* gene, obtained through FEL, FUBAR and MEME analysis

Domain	Total codons	FEL		FUBAR		MEME
		Codons under pervasive diversifying selection	Codons under pervasive purifying selection	Codons under pervasive diversifying selection	Codons under pervasive purifying selection	Codons under episodic diversifying selection
PYD	88 (1–88)	–	3, 15, 19, 26, 29, 63	–	3, 15, 26, 63	–
ID	218 (89–306)	234	113, 198, 301	91, 93, 105, 120, 218, 226, 233, 234, 250, 285	113, 198	234
HIN	203 (307–509)	344	320, 338, 357, 391, 420, 459	344, 398, 451, 458, 496	320, 357, 459	344, 451, 496

## Discussion

Different types of transcript variants of a gene may be produced due to exon-skipping events at transcription level. There are found three transcript variants of different lengths of *IFI16-L* gene in each *ruminant* under study. These transcript variants can translate to form PYD-only proteins (POP) and HIN-only proteins (HOP). The presence of these POPs and HOPs has also been mentioned in the earlier studies [15, 16]. It appears that the main transcript is maintained for common pathogen threats while variable length transcript variants may be required in the case of exposure to new pathogens. The alternative splicing of immune genes has also been reported to occur in a tissue-specific manner [51]. These splice forms increase the diversity of particular protein and this may be a possible adaptive mechanism by which the pathogenic counteraction can be neutralized [52–54]. It is also possible that some of the transcript variants (most probably POPs and HOPs) may act as negative regulators of *IFI16-L* signaling [55].

Nuclear Localization Signal (NLS) is the positively charged, lysine or arginine-rich peptide residues at either N-terminal or C-terminal of the protein, which helps in importing protein into the nucleus through the nuclear pore complex. The presence of N-terminal NLS is a feature of nuclear *PYHIN* proteins. This feature of *PYHINs* can help to sense DNA viruses (like *herpesviruses*) in the nucleus as their DNA remains covered by an envelope in the cytoplasm. *IFI16* sense viral DNA in the nucleus and the nuclear shuttling of *IFI16* in and out is modulated by acetylation/de-acetylation status of its (*IFI16*) NLS [56]. This indicates that *IFI16-L* gene in the ruminants might be playing a role in nuclear viral DNA sensing in localization dependent manner similar to *IFI16* in humans.

The HIN domain ML phylogram produced three clades of placental mammal sequences based on their HIN domain types i.e. HINa, HINb and HINc while the fourth clade of the marsupial sequence has HINd domain which aligns separately. The HIN-C type domain is the exclusive property of *AIM2-like* receptors [16]. The different types of HIN domains are previously reported for their different DNA binding abilities. These different types of HIN domains in mammalian lineages may have evolved for different types of DNA structures from different pathogens present in the niche of a particular lineage [10, 16].

On the other hand, the PYD domain phylogram produced two separate clades among placental mammals while the third clade is made of the marsupial sequence. Separate evolutionary history of *AIM2-like PYHIN* genes indicates a possible different role of *AIM2-like* PYD domain in the cellular physiology to the other types of PYD domains. *IFI16-L PYHIN* gene in the ruminants and cetaceans are quite similar, despite of differences in their habitats (land and marine respectively). In the phylogenetic tree of inter-domain linker region, all the sequences were aligned into single clade; which shows that inter-domains of *IFI16-L* genes are conserved in related species.

Eukaryotic cells are equipped with different types of DNA sensors to sense and respond against the different types of invading foreign DNA. In nature, hosts and pathogens have been co-evolving. Their interactions put selection pressure on each other which results in a diversifying selection. *IFI16-L PYHIN* genes have three distinct parts with totally different functions which are, the N-terminal PYD domain that homotypically binds and interacts with the PYD domain of adaptor protein *PYCARD* for downstream signaling, the C-terminal HIN domain which binds with pathogenic dsDNA and the inter-domain linker region which may play a role in localization of PYD and HIN domains for their proper functioning. Based on their different functions the nature of selection might be acting differently on these distinct parts.

The distribution of positively selected sites on HIN domain and inter-domain linker region is congruent with the previous study on primate *IFI16*. But in the case of PYD domain in ruminant *IFI16-L*, no positively selected sites were found however, three positively selected sites were reported on the PYD domain of primate *IFI16* [19]. At the interacting sites of the host and the pathogen proteins, there are more non-synonymous substitutions (dN) which drives the positive selection on these interacting residues. To evade the host sensing mechanism, the invading viral proteins target the ALRs as a counteract measure. The *pUL83* protein which is encoded by herpesvirus HCMV binds to the PYD domain of *IFI16* and blocks its oligomerization hence, inhibiting the downstream signaling process [57]. This interaction of viral *pUL83* protein might be the driving force for positive selection in the PYD domain of primate *IFI16*.

However, similar selection pressure in PYD domain appears absent in ruminant species. *HSV-1* encoded protein immediate early protein *ICP0,* in a way to evade host defense recognition mechanism, targets and binds to *IFI16.* It leads to its (*IFI16*) proteasome mediated degradation process which ultimately results in the loss of *IFI16* protein [58]. Evidence of positive selection pressure might help to evolve new host residues to prevent viral antagonists and subsequent host target modifications. The HIN domain is primarily involved in the binding of foreign DNA directly thus, it should have been under the influence of selective pressure, but there are also positively selected residues were found in the inter-domain region of *IFI16* and *IFI16-L PYHIN* genes of primates and ruminants respectively.

As we know that viral DNA sensing by *IFI16* is localization dependent, so the nuclear localization signal (NLS) might be the prime target of viral factors to interfere in the translocation of *IFI16-L*. Some part of NLS lies in the PYD domain and the rest resides in the inter-domain linker. The part of NLS that resides in the inter-domain has two positively selected sites which show that this NLS part is engaged in the conflicts with viral defense proteins. In this regard, further studies are required to fully understand the role of *IFI16-like PYHIN* genes in context to their foreign DNA binding capabilities. Pathogens are one of the strongest selective forces. The host PRRs is evolved to recognize special featured structures of pathogen called PAMPs. The purifying selection on the PYD domain may be compared to that of house-keeping genes that are essential for proper functioning of vital molecular machinery. Any mutation in these genes will be deleterious and will be eliminated. The purifying selection has important role in long term stability of biological structures by removing deleterious mutations [25, 59–61].

## Conclusion

*ALRs* of *PYHIN* family genes are primitive class of innate immune PRRs of pathogenic double stranded (dsDNA) found in (but not limited to) mammals. A single functional *PYHIN* gene i.e. *IFI16-like (IFI16-L)* gene is reported in the clade Ruminantia. *IFI16-L* has evolutionarily conserved orthology in Ruminantia and Cetacea which are quite similar clades. Because of its activity as an immune sensor just like human *IFI16*, it might be engaged in direct conflicts with pathogenic proteins. This phenomenon could put pressure on *IFI16-L* gene to evolve. *IFI16-L* protein has three distinct parts- the PYD domain, the HIN domain and the inter-domain linker region; to perform separate activities. Due to this, the nature of selection pressure also varies on these different parts.

In the current study, the selective pressure that acts on *IFI16-like PYHIN* genes in ruminants was investigated. The inter-domain region is influenced by maximum diversifying selection pressure which indicates its involvement in the interaction with pathogenic defense factors. The DNA binding HIN domain is also under the influence of diversifying selection pressure. This *IFI16-L* ALR encodes three transcript variants with complete ORFs. This may be an adaptive strategy to lame the viral defense. Also, some of these transcript variants may play a role in the regulation of its *(IFI16-L)* activities. Furthermore, research is needed to shed more light on the role of the *IFI16-like PYHIN* genes, particularly its transcript variants, in immune DNA sensing and the interactions of inter-domain linker region with various pathogenic proteins.

## Materials and methods

### Sample collection

We assembled blood [into heparinized vacuutainers (BD)] of four ruminant species from National Dairy Research Institute (NDRI) animal herd and local slaughterhouse, as follows: *water buffalo (Bubalus bubalis)*, *zebu cattle (Bos indicus)*, *goat (Capra hircus)*, *sheep (Ovis aries)*. Three sets of blood samples were collected as biological replicates, immediately PBMCs were separated; mixed with Trizol Reagent (Invitrogen, USA) and frozen into − 80 °C for further use.

### PCR, cloning and sequencing

Total RNA was extracted from 0.5 mL of collected blood samples (TRIzol reagent Method). The quality and quantity of RNA samples were determined using a NanoDrop 2000 spectrophotometer (Thermo Scientific, USA) and were further ascertained by agarose gel electrophoresis. One μg of each RNA sample was reverse transcribed using the RevertAid First Strand cDNA synthesis kit (Thermo Scientific, USA), by using random hexamer primers according to the manufacturer’s instructions. Oligonucleotide primers (Table 2) for full coding regions of *IFI16-L* genes were designed from the conserved regions of the predicted *IFI16-L* gene sequences of ruminants (XM_006060336, XM_863928, XM_012172466, XM_005701415) from GenBank. Around 100 ng of cDNA was used as the template for each amplification reaction containing 0.5 μL Advantage Taq Polymerase (Clontech, USA), 10 pmol of each *IFI16-L* primers in a total of 25 μL reaction mix. The amplified PCR products were cloned using InsTAclone PCR cloning kit (Thermo Scientific, USA) and transformed into XL1-Blue competent *E.coli* cells (Stratagene). The recombinant clones were subsequently screened through PCR. Sanger’s sequencing was performed with DTCS quick start cycle sequencing kit (Beckman Coulter) and run on GenomeLab™ GeXP Genetic Analysis System (Beckman Coulter).

**Table 2:** Primers used for gene amplification and sequencing

Gene	Primer name	Sequence (5′ – 3′)	T_m_ (°C)	Length (bp)
*IFI16-L*	IFI16FW (Full ORF)	AGCCAGCACTAAATCAAGGGTTTTGACTAGTC	62	32
	IFI16RV (Full ORF)	GCCAAGTTATAAAAGTTATAGATGCAAAATGGGACA	60	36
	IFI16FW2	GTGACACAGCCACCAAACCTAAGGAC	61	26
	IFI16FW3	GAGGCACATCCCTAATTTGAGAGATGGGG	63	29
	IFI16RV1	ATCTGGCCACTGCTCACAGTTGGG	61	24
*M13*	M13/pUC	GTAAAACGACGGCCAGT	47	17
	M13/pUCRV	CAGGAAACAGCTATGAC	45	17

### Immunofluorescence assay

Buffalo fetal fibroblasts were cultured for 24 h at 37 °C with 5% CO2 and the next day the cells were fixed with 4% paraformaldehyde for 30 min at 4 °C. Cells were incubated with the Anti-*IFI16* polyclonal antibody (SIGMA) at 37 °C for 1 h and afterward incubated with FITC-conjugated Anti-rabbit secondary antibodies under the same conditions. Dilution of both primary and secondary antibodies used was 1:50. The cells were further co-stained with 1 μg/mL of DAPI and examined under fluorescent microscope.

### Bioinformatics analysis

**Homologs** of *IFI16-L* gene were searched with NCBI BLASTn tool and UCSC-BLAT. Additional mammalian *PYHIN* nucleotide sequences were retrieved from NCBI Database for phylogenetic analysis (see Additional file 3). Amino acid sequences of these *ALRs* were obtained by translating the respective nucleotide sequences using the ExPASy Translate tool. Molecular weights and isoelectric points were calculated using the Compute pI/Mw tool [62]. Conserved domain architectures of *IFI16-L* protein sequences were determined with InterPro [63] and Conserved Domain Search Service [64]. We predicted a nuclear localization signal (NLS) in the N-terminal part of *IFI16-L ALRs* using the NLStradamus program [65], which is based on Hidden Markov models (HMMs) statistical approaches.

### Phylogenetic analysis

Nucleotide sequences of PYD domain, HIN-200 domain and inter-domain (Additional file 3) were aligned separately using the RevTrans 2.0 web server [34], which uses a scaffold of protein sequence alignment to construct the DNA multiple alignments of the coding DNA in order to reduce the signal to noise ratio. Divergent sequences which created alignments errors; were identified and removed manually. Phylogenetic analyses of PYD and HIN domains were conducted using “one-click” mode at *Phylogeny.fr* suite of programs [35, 66]. Translated amino acid sequences were given as input which was aligned using MUSCLE 3.8.31 and used Gblocks 0.91b filter to check ambiguous (poorly aligned and divergent) sequence regions. No ambiguous sequence was found in any of the alignments under study. A maximum likelihood tree was created using PhyML 3.0 with 100 bootstrap replicates and the tree was rendered using TreeDyn 198.3 software. Trees of the functional domains i.e. PYD and HIN, were re-rooted on *Sarcophilus harrisii* (marsupial), which is taken as an outgroup; while in the case of inter-domain phylogenetics tree, *Orcinus orca* (cetacean), is taken as an outgroup. All the nodes having bootstrap values of < 50% were collapsed. Trees were exported in the Newick format and visualized using iTOL online software [67].

To determine the selective force on *IFI16-L* gene in ruminants, nucleotide sequences of eight ruminants (*water buffalo, zebu cattle, goat, sheep, Taurus cattle, domestic yak, bison and Tibetan antelope*) were selected. The PYD domain, HIN domain and the inter-domain linker region nucleotide sequences were aligned separately with the RevTrans 2.0 utility and the codon sequences were subsequently used for selection pressure analysis. Amino acid sequence alignments of PYD domain, HIN domain and inter-domain region are provided in Additional file 4.

### Selective pressure analysis

Prior to selective pressure analysis, we checked all of the groups of nucleotide sequences for intragenic recombination. Recombination in sequences was tested with a difference of the sum of squares (DSS) method using a step size of 10 bp and a sliding window of 80 bp [36] as implemented in the software package TOPALi v2.5 [37]. Intragenic recombination was also assessed through GARD (Genetic Analysis for Recombination Detection) analyses [38, 39]. The significant topological incongruence of recombination breakpoint was tested using the Kishino–Hasegawa test (KH test) [68].

We used statistical tools from the HyPhy package [69] implemented in the Datamonkey web server [38, 70] to characterize the patterns of selection on the alignments. The Neighbour-Joining (NJ) tree, with HKY85 [71] nucleotide substitution model, was selected with the HyPhy model selection tool for each alignment. For the analysis with HyPhy softwares, the default set parameters of *p*-values/posterior probabilities were taken into account for each analysis. Maximum likelihood tests were performed utilizing the partitioning approach for robust inference of selection (PARRIS) method [47], which is an extension of the standard likelihood ratio test and can also better deal with recombining sequences. FEL (Fixed Effect Likelihood) and FUBAR (Fast Unbiased Bayesian AppRoximation) [42, 48] were utilized to identify sites evolving under pervasive diversifying and pervasive purifying selection pressures. MEME (Mixed Effects Model of Evolution) [40] was used to identify episodically diversifying sites. FEL is an ML based model which assumes constant selection pressure on each site. The FUBAR model estimates site-by-site ω values while MEME allows the distribution of ω to vary over sites and additionally from branch to branch. Significance was assessed by posterior probability > 0.9 (FUBAR) and *p*-value < 0.05 (MEME), p-value < 0.1 (FEL) and *p*-value < 0.1 (PARRIS).

## Additional files

Additional file 1: Diversity of *PYHIN* genes. Orthology in *PYHIN* genes is not conserved. The number and type of *PYHIN* genes varies in different mammalian orders. Here, the *PYHIN* genes and their associated domains from selected orders of classes Mammalia, Reptilia and Sarcopterygii (fish) have been presented. The residue lengths of PYD and HIN domains of all the animals are conserved. Drastic variation in the length of the inter-domain linker regions (shown in grey colour) can be observed among the various animals as well as among the different *PYHIN* genes within the same animal. The small HIN region within the *IFI16-L* from the elephant represents a rudimentary form of HINa domain. (JPG 345 kb)

Additional file 2: Tables containing PARRIS results of (a) PYD domain, (b) inter-domain and (c) HIN domain. (DOCX 17 kb)

Additional file 3: *PYHIN* sequences used for phylogenetic analysis. (DOCX 13 kb)

Additional file 4: Multiple sequence alignments of (a) PYD domain, (b) inter-domain and (c) HIN domain. (DOCX 101 kb)

## Abbreviations

AIM2: Absent in melanoma 2
ALRs: AIM2-like receptors
ALRs: AIM2-Like receptors
CADM3: Cell Adhesion molecule 3
cGAS: cyclic GMP-AMP Synthase
CLRs: C-type lectin receptors
CMS: Codon model selection
DAI: Z-DNA binding protein 1
DAMPs: Damage associated molecular receptors
dsDNA: double stranded DNA
DSS: Difference of the Sum of Squares
FUBAR: Fast unbiased bayesian approximation
GARD: Genetic Analysis of recombination detection
HCMV: Human cytomegalovirus
HIN-200: Hematopoietic interferon inducible nuclear protein with 200 amino acid repeat
HMM: Hidden markov model
HOP: HIN-only protein
HSV-1: Herpes simplex virus-1
IFI16: Interferon-γ inducible protein 16
IFI16-L: Interferon-γ inducible protein 16-like
KH Test: Kishino-hasegawa test
MEME: Mixed effect model of evolution
NJ: Neighbor joining
NLRs: NOD-like receptors
NLS: Nuclear localization signal
PAMPs: Pathogen associated molecular receptors
PARRIS: Partitioning approach for robust inference of selection
PBMCs: Peripheral blood mononuclear cells
POP: PYD-only protein
PRRs: Pattern recognition receptors
PYHIN: PYD and HIN-200 Domain containing
RLRs: RIG-1-like Receptors
SPTA1: Spectrin Alpha Chain 1
ssDNA: Single stranded DNA
TLRs: Toll-like receptors
