# Supplementary figures and images for: Nature of selection varies on different domains of IFI16-like PYHIN genes in ruminants
Source: BMC Evol Biol. 2019 Jan 17;19:26. doi: 10.1186/s12862-018-1334-7 (PMC6335826; doi:10.1186/s12862-018-1334-7)

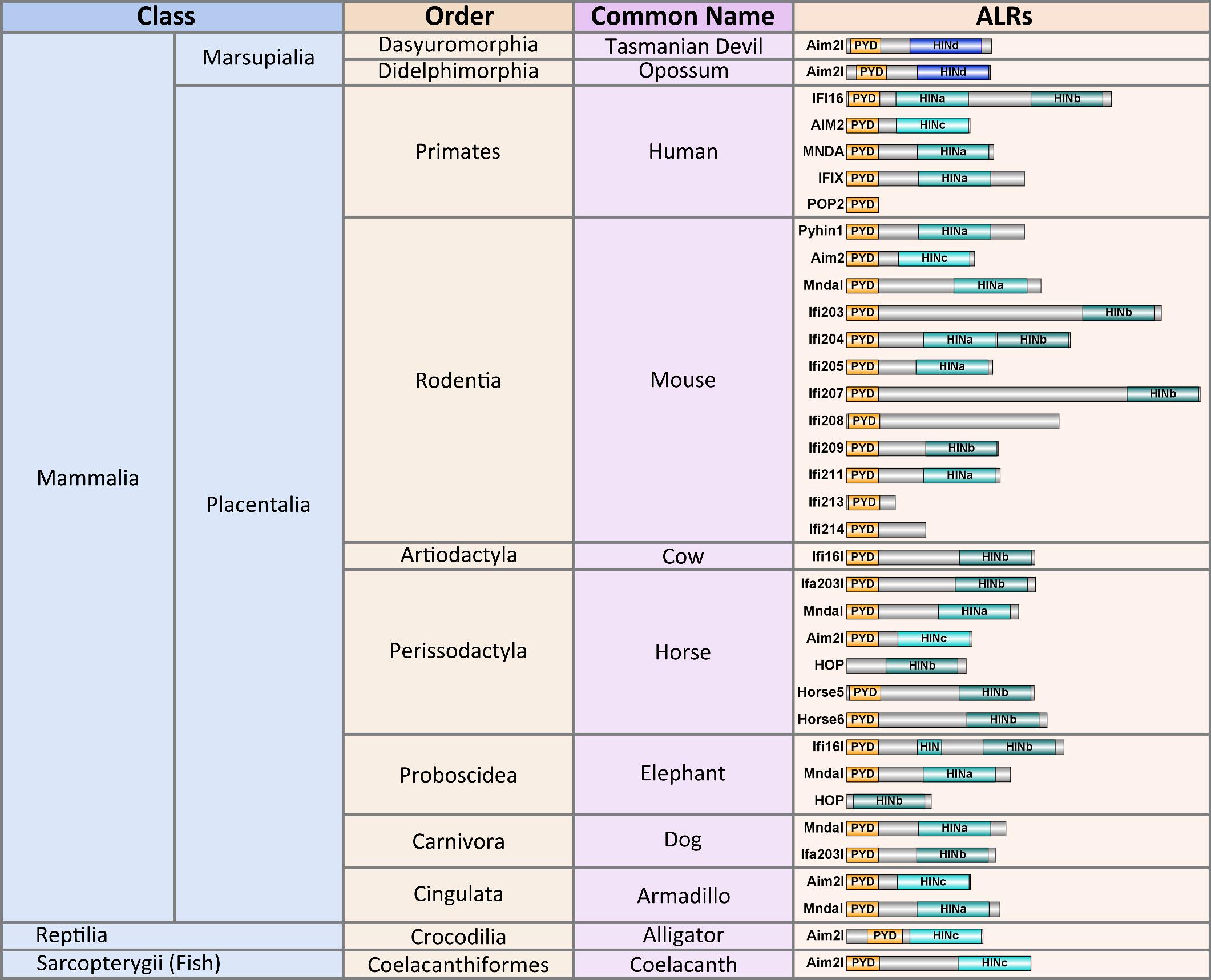

Supplement: Supplementary file 1 — Diversity of PYHIN genes. Orthology in PYHIN genes is not conserved. The number and type of PYHIN genes varies in different mammalian orders. Here, the PYHIN genes and their associated domains from selected orders of classes Mammalia, Reptilia and Sarcopterygii (fish) have been presented. The residue lengths of PYD and HIN domains of all the animals are conserved. Drastic variation in the length of the inter-domain linker regions (shown in grey colour) can be observed among the various animals as well as among the different PYHIN genes within the same animal. The small HIN region within the IFI16-L from the elephant represents a rudimentary form of HINa domain. (JPG 345 kb) [file 12862_2018_1334_MOESM1_ESM.jpg]
