# Supplementary material for: Nature of selection varies on different domains of IFI16-like PYHIN genes in ruminants
Source: BMC Evol Biol. 2019 Jan 17;19:26. doi: 10.1186/s12862-018-1334-7 (PMC6335826; doi:10.1186/s12862-018-1334-7)
Supplement: Supplementary file 2 — Tables containing PARRIS results of (a) PYD domain, (b) inter-domain and (c) HIN domain. (DOCX 17 kb) [file 12862_2018_1334_MOESM2_ESM.docx]

**Additional file 4: Tables containing PARRIS results of (a) PYD domain, (b) inter-domain and (c) HIN domain.**

**(a) PYD domain**

**Inferred rate distributions** for the null model (M1)

| \| **Synonymous rate** \| \| \| \| \| \| --- \| --- \| --- \| --- \| --- \| \| Rate Class \| 1 \| 2 \| 3 \| Summary \| \| dS \| 0.63 \| 2.03 \| 5.34 \| Mean : 1.000 \| \| Prob. \| 0.921 \| 0.000 \| 0.079 \| Std.Dev : 1.269 \| | \| **dN/dS ratio ω** \| \| \| \| \| --- \| --- \| --- \| --- \| \| Rate Class \| 1 \| 2 \| Summary \| \| ω \| 0.00 \| 1.00 \| Mean : 0.479 \| \| Prob. \| 0.521 \| 0.479 \| Std.Dev : 0.500 \| |
| --- | --- | --- | --- | --- | --- | --- | --- | --- | --- | --- | --- | --- | --- | --- | --- | --- | --- | --- | --- | --- | --- | --- | --- | --- | --- | --- | --- | --- | --- | --- | --- | --- | --- | --- | --- | --- | --- |

**Inferred rate distributions** for the alternative model (M2)

| \| **Synonymous rate** \| \| \| \| \| \| --- \| --- \| --- \| --- \| --- \| \| Rate Class \| 1 \| 2 \| 3 \| Summary \| \| dS \| 0.63 \| 2.61 \| 5.34 \| Mean : 1.000 \| \| Prob. \| 0.921 \| 0.000 \| 0.079 \| Std.Dev : 1.269 \| | \| **dN/dS ratio ω** \| \| \| \| \| \| --- \| --- \| --- \| --- \| --- \| \| Rate Class \| 1 \| 2 \| 3 \| Summary \| \| ω \| 0.00 \| 1.00 \| 1.59 \| Mean : 0.479 \| \| Prob. \| 0.521 \| 0.479 \| 0.000 \| Std.Dev : 0.500 \| |
| --- | --- | --- | --- | --- | --- | --- | --- | --- | --- | --- | --- | --- | --- | --- | --- | --- | --- | --- | --- | --- | --- | --- | --- | --- | --- | --- | --- | --- | --- | --- | --- | --- | --- | --- | --- | --- | --- | --- | --- | --- | --- |

**(b) Inter domain**

**Inferred rate distributions** for the null model (M1)

| \| **Synonymous rate** \| \| \| \| \| \| --- \| --- \| --- \| --- \| --- \| \| Rate Class \| 1 \| 2 \| 3 \| Summary \| \| dS \| 0.00 \| 1.08 \| 3.53 \| Mean : 1.000 \| \| Prob. \| 0.282 \| 0.626 \| 0.092 \| Std.Dev : 0.935 \| | \| **dN/dS ratio ω** \| \| \| \| \| --- \| --- \| --- \| --- \| \| Rate Class \| 1 \| 2 \| Summary \| \| ω \| 1.00 \| 1.00 \| Mean : 1.000 \| \| Prob. \| 0.033 \| 0.967 \| Std.Dev : -nan \| |
| --- | --- | --- | --- | --- | --- | --- | --- | --- | --- | --- | --- | --- | --- | --- | --- | --- | --- | --- | --- | --- | --- | --- | --- | --- | --- | --- | --- | --- | --- | --- | --- | --- | --- | --- | --- | --- | --- |

**Inferred rate distributions** for the alternative model (M2)

| \| **Synonymous rate** \| \| \| \| \| \| --- \| --- \| --- \| --- \| --- \| \| Rate Class \| 1 \| 2 \| 3 \| Summary \| \| dS \| 0.00 \| 0.99 \| 3.42 \| Mean : 1.000 \| \| Prob. \| 0.250 \| 0.644 \| 0.105 \| Std.Dev : 0.930 \| | \| **dN/dS ratio ω** \| \| \| \| \| \| --- \| --- \| --- \| --- \| --- \| \| Rate Class \| 1 \| 2 \| 3 \| Summary \| \| ω \| 0.90 \| 1.00 \| 1.69 \| Mean : 1.693 \| \| Prob. \| 0.000 \| 0.000 \| 1.000 \| Std.Dev : 0.000 \| |
| --- | --- | --- | --- | --- | --- | --- | --- | --- | --- | --- | --- | --- | --- | --- | --- | --- | --- | --- | --- | --- | --- | --- | --- | --- | --- | --- | --- | --- | --- | --- | --- | --- | --- | --- | --- | --- | --- | --- | --- | --- | --- |

**(c) HIN domain**

**Inferred rate distributions** for the null model (M1)

| \| **Synonymous rate** \| \| \| \| \| \| --- \| --- \| --- \| --- \| --- \| \| Rate Class \| 1 \| 2 \| 3 \| Summary \| \| dS \| 0.63 \| 2.44 \| 6.72 \| Mean : 1.000 \| \| Prob. \| 0.872 \| 0.096 \| 0.032 \| Std.Dev : 1.171 \| | \| **dN/dS ratio ω** \| \| \| \| \| --- \| --- \| --- \| --- \| \| Rate Class \| 1 \| 2 \| Summary \| \| ω \| 0.74 \| 1.00 \| Mean : 1.000 \| \| Prob. \| 0.000 \| 1.000 \| Std.Dev : 0.000 \| |
| --- | --- | --- | --- | --- | --- | --- | --- | --- | --- | --- | --- | --- | --- | --- | --- | --- | --- | --- | --- | --- | --- | --- | --- | --- | --- | --- | --- | --- | --- | --- | --- | --- | --- | --- | --- | --- | --- |

**Inferred rate distributions** for the alternative model (M2)

| \| **Synonymous rate** \| \| \| \| \| \| --- \| --- \| --- \| --- \| --- \| \| Rate Class \| 1 \| 2 \| 3 \| Summary \| \| dS \| 1.00 \| 1.69 \| 2.26 \| Mean : 1.000 \| \| Prob. \| 1.000 \| 0.000 \| 0.000 \| Std.Dev : 0.000 \| | \| **dN/dS ratio ω** \| \| \| \| \| \| --- \| --- \| --- \| --- \| --- \| \| Rate Class \| 1 \| 2 \| 3 \| Summary \| \| ω \| 1.00 \| 1.00 \| 12.39 \| Mean : 1.602 \| \| Prob. \| 0.377 \| 0.570 \| 0.053 \| Std.Dev : 2.548 \| |
| --- | --- | --- | --- | --- | --- | --- | --- | --- | --- | --- | --- | --- | --- | --- | --- | --- | --- | --- | --- | --- | --- | --- | --- | --- | --- | --- | --- | --- | --- | --- | --- | --- | --- | --- | --- | --- | --- | --- | --- | --- | --- |
