# Supplementary material for: Nature of selection varies on different domains of IFI16-like PYHIN genes in ruminants
Source: BMC Evol Biol. 2019 Jan 17;19:26. doi: 10.1186/s12862-018-1334-7 (PMC6335826; doi:10.1186/s12862-018-1334-7)
Supplement: Supplementary file 3 — PYHIN sequences used for phylogenetic analysis. (DOCX 13 kb) [file 12862_2018_1334_MOESM3_ESM.docx]

**Additional file2.TableS2. All mammalian PYHIN sequences used for phylogenetic analysis of PYD and HIN domains. For Phylogenetic reconstruction of inter-domain (ID) linker region, only bold sequences were included.**

| Sr. No. | Taxonomic Order | Animal Name | Gene Name | Domain type | Gene ID |
| --- | --- | --- | --- | --- | --- |
| 1. | Primate | *Homo sapiens* | AIM2 | PYD-HIN | NM001206567 |
|  |  |  | IFI16 | PYD-HIN-HIN | NM004833 |
|  |  |  | MNDA | PYD-HIN | NM002432 |
|  |  |  | IFIX | PYD-HIN | NM152501 |
| 2. | Rodentia | *Mus musculus* | AIM2 | PYD-HIN | NM001013779 |
|  |  |  | MNDA | PYD-HIN | XM017321420 |
|  |  |  | MNDAl | PYD-HIN | NM001170853 |
|  |  |  | IFI203 | PYD-HIN | NM001302649 |
|  |  |  | IFI204 | PYD-HIN-HIN | NM008329 |
|  |  |  | IFI205 | PYD-HIN | NM172648 |
|  |  |  | IFI207 | PYD-HIN | NM001204910 |
|  |  |  | IFI211 | PYD-HIN | NM001033450 |
| **3.** | **Artiodactyla** | ***Bubalus bubalis*** | **IFI16l** | **PYD-HIN** | KX931085 |
|  |  | ***Bos indicus*** | **IFI16l** | **PYD-HIN** | KX931088 |
|  |  | ***Bos taurus*** | **IFI16l** | **PYD-HIN** | XM863928 |
|  |  | ***Bos mutus*** | **IFI16l** | **PYD-HIN** | XM014478589 |
|  |  | ***Capra hircus*** | **IFI16l** | **PYD-HIN** | KX931091 |
|  |  | ***Ovis aries*** | **IFI16l** | **PYD-HIN** | KX931094 |
|  |  | ***Pantholops hodgsonii*** | **IFI16l** | **PYD-HIN** | XM005957402 |
|  |  | ***Bison bison*** | **IFI16l** | **PYD-HIN** | XM010846843 |
|  |  | *Lipotes vexillifer* | IFI16l | PYD-HIN | XM007445583 |
|  |  | *Orcinus orca* | IFI16l | PYD-HIN | XM012538519 |
| 4. | Perissodactyla | *Equus asinus* | AIM2l | PYD-HIN | XM014848305 |
| 5. | Carnivora | *Ailuropoda melanoleuca* | MNDA | PYD-HIN | XM011234808 |
| 6. | Proboscida | *Loxodonta africana* | MNDAl | PYD-HIN | XM010594862 |
| 7. | Sirenia | *Trichechus manatus* | MNDAl | PYD-HIN | XM012559745 |
| 8. | Pholidota | *Manis javanica* | AIM2 | PYD-HIN | XM017656146 |
| 9. | Dasyuromorphia | *Sarcophilus harrisii* | AIM2l | PYD-HIN | XM012548018 |
