# Supplementary material for: Nature of selection varies on different domains of IFI16-like PYHIN genes in ruminants
Source: BMC Evol Biol. 2019 Jan 17;19:26. doi: 10.1186/s12862-018-1334-7 (PMC6335826; doi:10.1186/s12862-018-1334-7)
Supplement: Supplementary file 4 — Multiple sequence alignments of (a) PYD domain, (b) inter-domain and (c) HIN domain. (DOCX 101 kb) [file 12862_2018_1334_MOESM4_ESM.docx]

**Additional file 3: Multiple sequence alignments of (a) PYD domain, (b) inter-domain and (c) HIN domain.**

**(a) PYD domain**


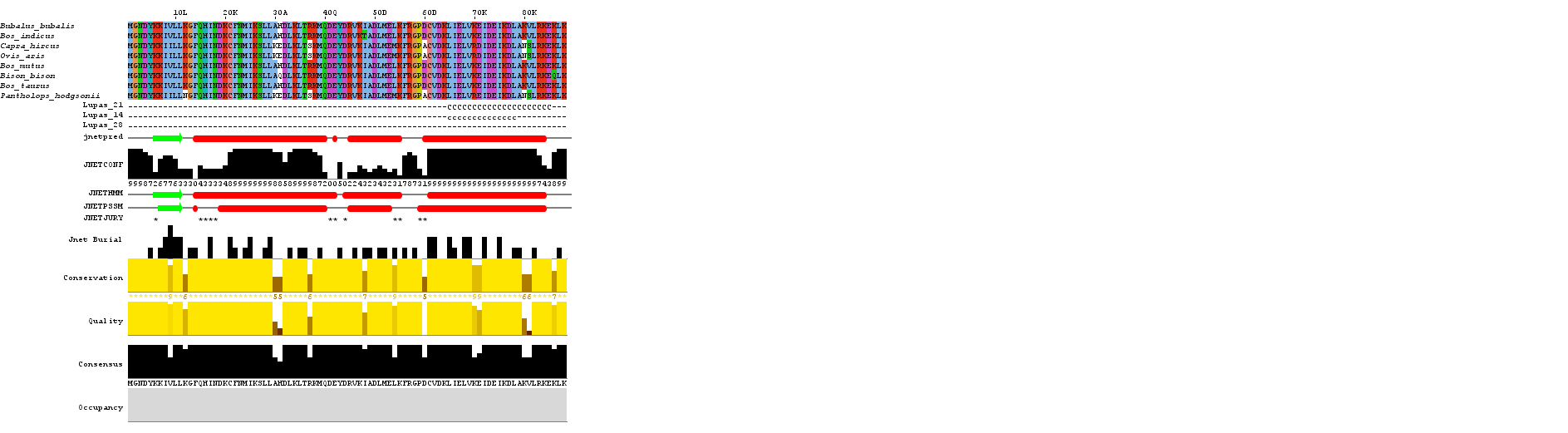


**(b) Inter domain**

**
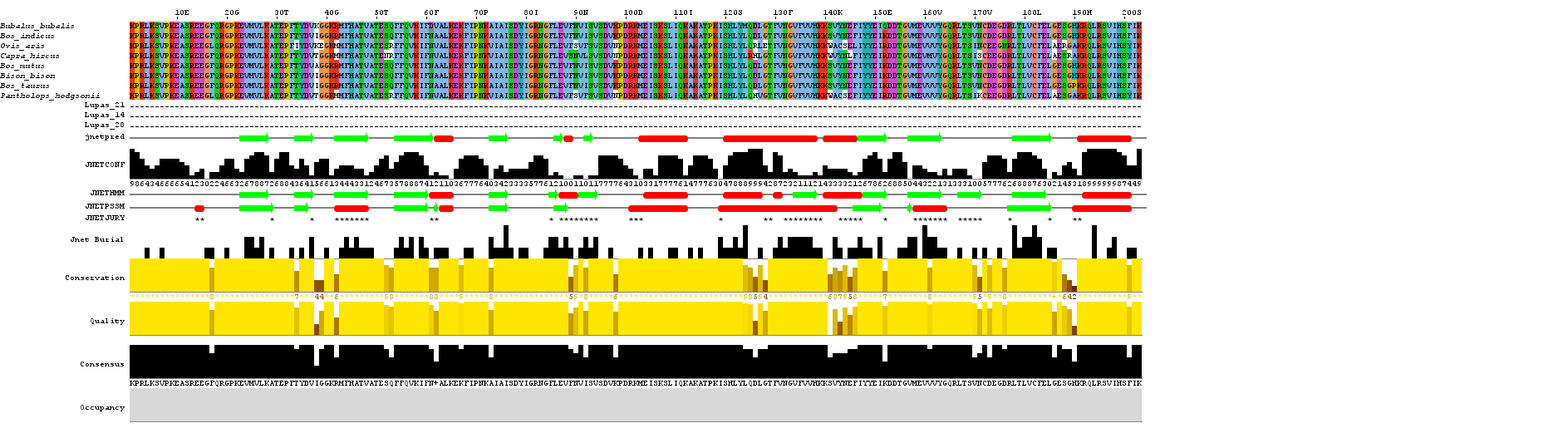
**

**(b) HIN domain**

**
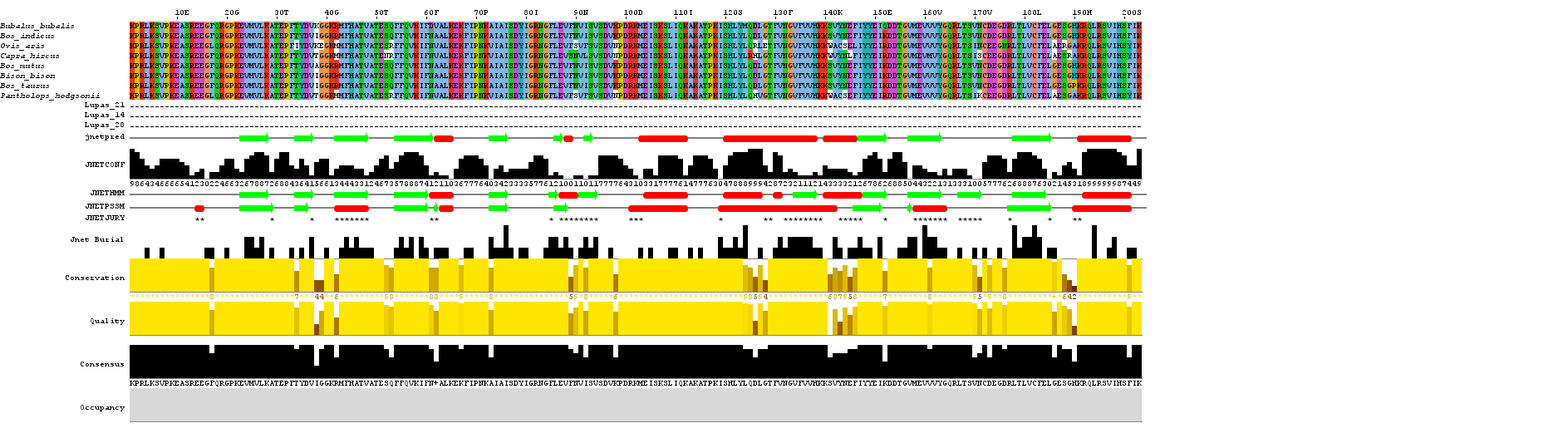
**
